# Alexithymia in eating disorders: Systematic review and meta-analyses of studies using the Toronto Alexithymia Scale

**DOI:** 10.1016/j.jpsychores.2017.06.007

**Published:** 2017-08

**Authors:** Heather Westwood, Jess Kerr-Gaffney, Daniel Stahl, Kate Tchanturia

**Affiliations:** a King's College London, Institute of Psychiatry, Psychology and Neuroscience, Psychological Medicine, London, UK; b King's College London, Institute of Psychiatry, Psychology and Neuroscience, Department of Biostatistics, London, UK; c South London and Maudsley NHS Trust, National Eating Disorders Service, Psychological Medicine Clinical Academic Group, UK; d Ilia State University, Tbilisi, Georgia

**Keywords:** Eating disorder, Alexithymia, Meta-analysis, Systematic review, Emotion recognition

## Abstract

**Objective:**

The aim of this review was to synthesise the literature on the use of the Toronto Alexithymia Scale (TAS) in eating disorder populations and Healthy Controls (HCs) and to compare TAS scores in these groups.

**Method:**

Electronic databases were searched systematically for studies using the TAS and meta-analyses were performed to statistically compare scores on the TAS between individuals with eating disorders and HCs.

**Results:**

Forty-eight studies using the TAS with both a clinical eating disorder group and HCs were identified. Of these, 44 were included in the meta-analyses, separated into: Anorexia Nervosa; Anorexia Nervosa, Restricting subtype; Anorexia Nervosa, Binge-Purge subtype, Bulimia Nervosa and Binge Eating Disorder. For all groups, there were significant differences with medium or large effect sizes between the clinical group and HCs, with the clinical group scoring significantly higher on the TAS, indicating greater difficulty with identifying and labelling emotions.

**Conclusion:**

Across the spectrum of eating disorders, individuals report having difficulties recognising or describing their emotions. Given the self-report design of the TAS, research to develop and evaluate treatments and clinician-administered assessments of alexithymia is warranted.

## Highlights

• Individuals across the spectrum of eating disorders have difficulties identifying and describing emotion
• Studies examining alexithymia in individuals with Binge Eating Disorder and those recovered from eating disorders are lacking
• Research on alexithymia would benefit from the development of clinically-based assessment tools

## 1 Introduction

Alexithymia, meaning literally “no words for mood” [1] was first coined in the 1970s to define an inability to describe and/or recognise one's own emotions. Since then, research has focused on both understanding alexithymia and on measuring it in both clinical and general populations. Alexithymia is known to be present in several psychiatric disorders, including depression [2]; Obsessive-Compulsive Disorder [3]; Schizophrenia [4]; Post-Traumatic Stress Disorder [5]; Autism Spectrum Disorder [6] and eating disorders (EDs) [e.g., 7]. While alexithymia is described as a stable personality trait [8] it correlates highly with symptoms of both depression and anxiety and may be a predisposing factor for the development of other psychopathologies [9]. What's more, alexithymia is thought to underlie emotional difficulties in individuals with eating disorders [10] and has been implicated in both the development and maintenance of EDs [11]. It is also related to poorer treatment outcome, making it a relevant treatment target [12].

Prevalence estimates of alexithymia within the general population, as measured by the twenty-item Toronto Alexithymia Scale [TAS-20; 13], range from 5.2 to 18.8%, with a prevalence of 18% being reported in a British undergraduate sample [14]. In this study, alexithymia was found be to be more prevalent in females than in males. Alexithymia is also associated with higher levels of sub-clinical disordered eating in undergraduate females [15], mirroring what has been found in ED populations [16], [17], [18].

One of the main focuses of alexithymia research has been how to effectively measure the concept. The development of the TAS-20 [13] resulted in increased interest in this field as it provides an efficient way to measure alexithymia, allowing for comparability across clinical groups [9]. The TAS-20 is a brief, self-report measure on which participants rate their level of agreement to statements on a five-point Likert scale, yielding a total score as well as subscale scores designed to measure: difficulty identifying feelings (DIF); difficulty describing feelings (DDF) and externally-oriented thinking (EOT). The maximum possible score on the TAS-20 is 100 with a score of 61 or above indicative of high levels of alexithymia [19]. The TAS-20 demonstrates good reliability and factorial validity [20], [21]. Despite the TAS being widely used, normative data for ED populations have not yet been reported. Synthesising studies comparing scores on the TAS in ED groups with Healthy Controls (HCs) would therefore aid comparison between existing and future studies.

The TAS-20 has been criticised for not measuring a universal alexithymia construct but perhaps instead measuring concepts such as social shame [22], negative emotional expressivity [23] or negative affect [24]. The factor structure of the TAS-20 may also vary across samples [25], highlighting the need to use the TAS-20 in combination with other measures of alexithymia [26]. Disagreement exists over exactly which constructs instruments are measuring and there is still a need for reliable, objective measures of alexithymia for use across psychiatric populations. Determining whether individuals with EDs consistently score higher on the TAS than controls would be useful for future research, aiming to develop new ways of measuring alexithymia in this population.

In a critical review of the literature on alexithymia in EDs, Nowakowski, McFarlane [18] report that individuals with EDs consistently report higher levels of alexithymia on the TAS than controls. However, as this review did not include meta-analysis of studies, it is not known whether the effect size is the same across the spectrum of EDs, e.g., in Anorexia Nervosa (AN), Bulimia Nervosa (BN) or Binge ED (BED), or whether a particular diagnosis is associated with higher levels of alexithymia. Nowakowski, McFarlane [18] report that individuals with EDs score higher on two of the TAS-20 subscales: DDF and DIF but not on EOT. Performing meta-analyses of subscale scores will help synthesise this literature further and to determine whether significant differences exist between groups on all sub-scale scores.

### 1.1 Aims of the study

This review aimed to synthesis the literature on the use of the Toronto Alexithymia Scale to assess alexithymia across the spectrum of eating disorder and to compare total and sub-scale scores on the Toronto Alexithymia Scale between eating disorder diagnoses.

## 2 Method

The systematic review and meta-analysis was conducted according to the PRISMA statement [27]. The quality of each study was assessed using the Clinical Appraisal Skills Programme checklist for case–control studies [28]. The tool consists of 11 questions, which yield a mixture of ‘yes’, ‘no’ and more qualitative answers. In this review, extra questions were added to more fully appraise the specific qualities of studies addressing alexithymia in EDs. These included whether confounding variables were accounted for in analysis and whether association between the TAS scores and other psychopathologies was examined. To calculate an overall quality rating, several questions were split into sub questions and a score of 1 was awarded for every ‘yes’ answered, with a maximum possible score of 17. The quality rating for each study is shown in Table 1.

**Table 1:** Characteristics of studies.

Author	Year	Group	N	Mean (SD) age	Mean (SD) BMI	Mean(SD) illness duration	HCs matched by	TAS version	Recruitment source	Diagnostic tool	Co-morbidities assessed	Quality score
Abbate-Daga et al. [50]	2015	AN	61	22.96 (5.46)	15.64 (1.57)	4.84(4.08)	Gender	TAS-20	Inpatient, day-patient	SCID-I	Depression, anxiety	15
		HC	59	24.50 (3.23)	20.69 (1.75)				University			
Adenzato et al. [51]	2012	AN	30	19.73 (6.06)	15.06 (1.74)	3.63 (5.27)	Age, education	TAS-20	ED unit	SCID for DSM IV	Depression	15
		HC	32	20.47 (2.72)	20.21 (1.45)							
Aloi et al. [48]	2017	BED	22	43.8 (10.07)	36.9 (4.2)			TAS-20	Obesity outpatient service	BED Clinical Interview	Depression	15
		HC (obese)	20	50.6 (8.6)	38.2 (6.5)							
Amianto et al. [47]	2016	AN	53	24.56 (8.32)	15.75 (1.62)		Gender, age range	TAS-20	Outpatient ED service	SCID-I	Depression, general psychopathology	15
		BN	71	28.19 (8.80)	21.96 (2.47)							
		HC	80	23.28 (0.63)	21.95 (2.46)				University database			
Bomba et al. [52]	2014	AN	60	15.43 (1.63)	15.51 (2.24)	11.2(8.75)*	Age	TAS-20	Inpatient, outpatient (child mental health department)	EDI-3	Depression	14
		HC	60	15.73 (1.88)	20.06 (2.17)				After-school youth centre			
Bourke et al. [53]	1992	AN	48	24.7 (6.3)	84.5 (12.1)% ABW	8.5	Age, education	TAS-26	ED unit		General psychopathology (CCEI)	13
		HC	30	26.8 (4.1)	98.8 (9.9)% ABW				Hospital staff			
Bydlowski et al. [44]	2005	AN	33	19 (2.2)		2.0 (1.9)	Age, education, SES	TAS-26	Psychiatric department patients	MINI	Depression, anxiety	16
		BN	37									
		HC	70	19.3 (1.9)					University			
D'Agata et al. [54]	2015	AN	21	21 (± 5)	16.1 (0.9)	> 2 years		TAS-20	ED centre	SCID for DSM-IV-R	Depression, anxiety	14
		BN	18	22 (± 5)	22 (2.3)							
		HC	17	23 (± 4)	21.5 (2.3)				Local advertisement			
Dapelo et al. [35]	2015	AN	20	28.85 (9.75)	15.59 (1.83)	11.55 (11.26)		TAS-20	Ed services, advertisement on Beat	SCID-I	Depression, anxiety	15
		BN	20	26.85 (6.75)	22.15 (3.02)	7.95 (6.46)						
		HC	20	26.40 (7.60)	22.47 (2.68)				Local community			
Dapelo et al. [55]	2016	AN	35	27.54 (8.36)	15.33 (1.74)	10.54 (9.16)	Age, education	TAS-20	Ed services (inpatients, outpatients), advertisement on Beat	SCID-I	Depression, anxiety, OCD	14
		HC	42	26.98 (7.55)	22.53 (2.63)				Local community, university			
de Groot et al. [56]	1995	BN	31	26.0 (5.8)	101.4 (18.1)% ABW	8.4 (6.1)		TAS-26	Day hospital programme	Clinical interview	Depression	14
		HC	20	30.9 (5.4)	100.1 (13.0%)% ABW				Nurses			
De Panfilis et al. [38]	2003	ED (AN, BN, BED)	64	32.2 (11.5)	AN = 16.65 (1.6), BN = 22.3 (5.6), BED = 34.6 (6.2)			TAS-20	Outpatients	SCID-I		14
		HC	68	29.8 (8.9)	21.4 (1.3)				University			
de Zwaan et al. [57]	1996	AN	22	22.2 (4.7)	14.9 (1.7)			TAS-20	Psychiatric department patients	SCID	Depression	14
		BN	18	23.9 (4.1)	20.7 (2.7)							
		HC	32	24.0 (3.6)	22.3 (3.6)				University			
de Zwaan et al. [58]	1995	BED	83	39.3 (7.0)	36.2 (3.9)			TAS-26	Eating disorder programme	QEWP	Depression	15
		HC (obese)	99	41.1 (8.9)	36.3 (4.5)							
Deborde et al. [59]	2008	ED (AN,BN)	47	22 (5.8)				TAS-20		MINI		7
		HC	253	25 (9.0)					University students & staff			
Eizaguirre et al. [43]	2004	ANR	25	21.08 (7.39)	16.96 (1.13)	46.80 (53.30)*	“Sociodemographic characteristics”	TAS-20	Referral from ED association		Depression, anxiety, general health	15
		ANBP	44	20.77 (4.74)	17.02 (0.99)	41.66 (40.16)*						
		BN	82	23.01 (5.35)	22.19 (3.44)	58.72 (48.33)*						
		HC	43	21.51 (5.97)	22.30 (2.36)							
Guttman & Laporte [60]	2002	ANR	34	22 (6)				TAS-20	Current and former outpatients	SCID for DSM-III-R	General psychopathology (SCL-90-R)	15
		BPD	35	32 (6)					Referral from therapists	DIB-R		
		HC	33	21 (5)					Public advertisement			
Jimerson et al. [61]	1994	BN	20	25 (4)	98 (8)% ABW		Age, weight	TAS-26	Outpatients, community advertisement	DSM-III-R	Depression, anxiety	15
		HC	20	24 (4)	98 (8)% ABW				Community advertisement, universities			
Kessler et al. [62]	2006	AN	48	22.9 (7.7)	16.3 (1.9)		Age	TAS-26	Inpatients		General psychopathology (SCL-90-R)	14
		BN	31	25.5 (8.4)	21.6 (4.1)							
		HC	78	22.8 (5.8)					Student nurses			
Kühnpast et al. [63]	2012	BN	13	24.6 (7.1)	21.7 (3.7)	8.1 (7.7)	Age, education BMI,	TAS-20	ED units	SCID-I	Depression, anxiety	12
		HC	13	25.4 (3.2)	21.6 (2.0)							
Lule et al. [64]	2014	AN	15	16.2 (1.26)	17.07 (1.44)		Age, gender, education	TAS-26	Inpatients, outpatients		Depression, anxiety, general psychopathology (YSR & CBCL)	13
		HC	15	16.5 (1.09)	21.06 (1.57)				Schools			
Marchesi et al. [24]	2014	ED (AN, BN, BED)	52	43.5 (12.5)	< 16 excluded		Age, gender	TAS-20	Outpatients	SCID for DSM-IV	Anxiety, depression	15
		HC	78	41.2 (11.8)					University, hospital staff			
Matsumato et al. [65]	2015	AN	22	25.77 (6.26)	15.87 (2.62)	7.24 (6.47)	Age	TAS-20	Hospital	DSM-IV	Anxiety, depression, OCD	14
		BN	36	25.94 (5.81)	19.76 (2.38)	7.15 (5.80)						
		HC	51	23.82 (5.58)	20.99 (1.71)				Local advertisement, university			
Miyake et al. [66]	2012	AN	30	27.2 (6.5)	15.4 (1.7)	6.17 (4.0)		TAS-20	Outpatients	SCID for DSM-IV		15
		HC	20	25.6 (4.6)	19.3 (1.9)				Community advertisement			
Montebarocci et al. [67]	2006	AN	18	19.3 (1.6)	14.22 (1.70)		Age, education	TAS-20	Day hospital programme	SCID-I	Depression, anxiety	13
		BN	16	18.4 (2.3)								
		HC	18	19.1 (1.8)								
Nandrino et al. [68]	2012	ANR	16	21.7 (4.2)	15.5 (1.03)		Age, gender, education	TAS-20	Inpatients		Depression, anxiety	12
		HC	20	20.45 (1.73)	21.2 (2.2)				University			
Nandrino et al. [69]	2006	ANR	25	23.7 (6.4)	15 (1.26)	5.96 (4.4)	Age, gender, education	TAS-20	Inpatients		Depression, anxiety	12
		HC	25	23.56 (4.87)	21.54 (1.92)				University			
Parling et al. [45]	2010	AN	35	21.6 (4.6)	18.98 (2.2)		Age	TAS-20	Former inpatients, now outpatients		Depression, anxiety	16
		HC	34	22.6 (5.3)	22.02 (2.2)				High school students, hospital staff			
Pinaquy et al. [70]	2003	BED	40	38.1	36.8		Age, BMI, education, SES	TAS-20	Obesity outpatient service	QEWP-R	Depression, anxiety, stress	15
		HC (obese)	129	40.4	35.7							
Pollatos et al. [71]	2008a	ANR	12	23.86 (4.25)	16.34 (1.14)	3.7 (3.2)	Age, gender, education	TAS-20	Self-help groups	SCID-I	Depression, anxiety	11
		HC	12	22.39 (4.78)	22.95 (4.52)				Students			
Pollatos et al. [72]	2008b	AN	28	21.43 (2.38)	16.59 (1.16)	2.5 (3.2)	Age, gender, education	TAS-20	Self-help groups	SIAB-S	Depression, anxiety	12
		HC	28	22.39 (4.78)	21.49 (4.28)				Students			
Pollatos & Georgiou [73]	2016	BN	23	24.0 (7.2)	20.9 (3.4)	5.9 (3.7)	Age, education, BMI, gender	TAS-20	Counselling ED units	SCID-I	Depression, anxiety	11
		HC	23	25.1 (3.2)	22.0 (2.5)							
Rommel et al. [41]	2013	ANR	25	19.9 (3.1)	14.83 (1.66)	2.97 (2.13)		TAS-20	Inpatients			15
		ANBP/BN	19	20.76 (3.76)	18.31 (3.49)	4.81 (3.43)						
		HC	37	20.49 (3.04)	20.37 (2.47)				Students			
Schmidt et al. [74]	1993	BN	93	23.8 (5.1)	21.3 (2.6)			TAS-26	ED clinic			12
		ANR	55	23.6 (5.7)	15.8 (2.3)							
		ANBP	25	22.4 (5.9)	17.6 (2.0)							
		HC female	48	21 (3.0)					University students			
		HC male	47	21/2 (2.5)								
Sexton et al. [75]	1998	ANR	15	25.7 (12.2)	14.9 (2.8)			TAS-26	Inpatients	SCID for DSM-III-R	Depression	13
		ANBP	16	27.7 (9.1)	16.1 (2.8)							
		BN	22	25.4 (8.3)	22.7 (4.6)							
		HC high levels of dietary restraint	14	33.2 (8.4)	22.3 (1.8)				Local advertisement			
Speranza et al. [42]	2004	AN	149	20.8 (5.2)			Age, gender, professional status	TAS-20	Inpatients, outpatients	MINI	Depression	14
		BN	84	23.1 (5.0)								
		HC	518						Advertisement in nursing schools & medical faculties			
Speranza et al. [76]	2005	ANR	105	20.4 (5.3)	15.0 (1.8)		Age, SES	TAS-20	Inpatients, outpatients	MINI	Depression	15
		ANBP	49	21.6 (4.9)	15.9 (2.1)							
		BN	98	23.5 (5.7)	21.2 (3.9)							
		HC	279	21.8 (5.3)	21.1 (2.8)				Nursing schools & medical facilities			
Strigo et al. [40]	2013	REC AN	12	29.7 (6.8)	21.9 (1.65)	10.7 (8.17)		TAS-20		SCID-I	Depression, anxiety	12
		HC	10	24.8 (6.1)	21.9 (0.73)							
Sureda et al. [77]	1999	BN	35	23.4 (5.5)		5.47 (4.07)	Age, education	TAS-26	Outpatients			15
		HC	35	23.37 (6.1)					Hospital staff			
Svaldi et al. [78]	2010	BED	27	42.7 (11.6)	36.7 (3.89)			TAS-20			Depression	14
		HC (obese)	25	38.3 (13.8)	33.8 (6.53)							
Taylor et al. [79]	1996	AN	48	24.7 (6.3)	84.5 (12.1)% ABW		Age, education	TAS-20	ED clinic			13
		HC	30	26.8 (4.1)	98.9 (2.2)% ABW				Hospital staff			
		HC female students	118	21.7 (3.0)			Unmatched		Physical education majors			
		HC male students	116	21.5 (3.4)								
Tchanturia et al. [49]	2012	AN	72	25.7 (7.5)	14.5 (1.8)	9.6 (7.0)		TAS-20	Inpatients, outpatients	SCID	Depression, anxiety	15
		BN	19	31.0 (11.7)	21.0 (2.1)	11.1 (9.4)			Outpatients			
		REC AN	14	25.2 (8.7)	21.1 (1.9)	3.8 (2.6)			Research volunteer database			
		HC	43	26.5 (8.8)	21.6 (1.7)				Local advertisements			
Torres et al. [80]	2011	AN	80	19.21 (5.39)	15.27 (1.49)	Median 2 years	Age, education	TAS-20	Hospitals, private clinics	IDED-IV	Depression, anxiety	14
		HC	80	19.2 (4.76)	21.08 (1.39)				High school and university students			
Torres et al. [46]	2015	ANR	52	18.19 (5.01)	15.41 (1.55)	37.9 (45.61)*	Age, education, SES	TAS-20	Inpatients, outpatients	IDED-IV	Depression	16
		ANBP	28	21.11 (5.65)	14.99 (1.39)							
		HC	80	19.2 (4.76)	21.08 (1.39)				High school and university students			
Troop et al. [81]	1995	ANR	29	25.5 (6.5)	16.2 (2.6)			TAS-26	ED clinic			13
		ANBP	15	23.3 (5.3)	17.7 (1.8)							
		BN	83	24.5 (5.6)	21.3 (2.7)							
		HC	79	20.6 (3.1)					Psychology students			
Zeeck et al. [82]	2011	BED	20	39.3 (12.7)	42.8 (6.0)		Age, education	TAS-20	Outpatients	Structured interview	Depression, general psychopathology (SCL-90-R)	14
		HC (obese)	23	45.4 (11.3)	41.1 (6.7)							
Zonnevylle-Bender et al. [83]	2002	AN	16	14.9	15.4		Age, education, SES	TAS-20	ED unit			14
		BN	8	16.8	21.3							
		EDNOS	6	16.2	20.3							
		HC	31	16.1	19.5				Schools			
Zonnevylle-Bender et al. [84]	2004	ANR	48	15.5 (1.1)			Age	TAS-20	Outpatients	EDE	Depression	15
		HC	48	15.4 (1.3)					High school students			

ABW = Average Body Weight; AN = Anorexia Nervosa; ANBP = Anorexia Nervosa, Binge-Purge subtype; ANR = Anorexia Nervosa, Restrictive subtype; BED = Binge Eating Disorder; BMI = Body Mass Index; BN = Bulimia Nervosa; CCEI = Crown Crisp Experimental Index; DSM = Diagnostic and Statistical Manual of Mental Disorders; EDE = Eating Disorder Examination; EDI-3 = Eating Disorder Inventory, 3rd edition; HC = Healthy Control; IDED = Interview for Diagnosis of Eating Disorders; MINI = International Neuropsychiatric Interview; OCD = Obsessive-Compulsive Disorder; REC AN = Recovered from Anorexia Nervosa; SCID = Structured Clinical Interview for DSM disorders; SCL-90-R = Symptom Checklist 90, Revised; SES = Socioeconomic Status; TAS-20 = Twenty-item Toronto Alexithymia Scale; TAS-26 = Twenty-six Item Toronto Alexithymia Scale; QEWP = Questionnaire of Eating and Weight Patterns; * = mean age given in months.

### 2.1 Eligibility criteria

Studies using either the TAS-20 or TAS-26 with both a clinical ED population and HCs were included in the review. Inclusion criteria were: 1) full text available in English; 2) reporting mean and standard deviation TAS total scores for both groups; 3) published in a peer-reviewed journal.

### 2.2 Information sources and search

The electronic databases PsychInfo, Scopus, Pubmed and Web of Science were searched systematically for papers up to and including May 2017. The search terms were either Anorexia Nervosa, Bulimia Nervosa or ED and alexithymia or Toronto Alexithymia Scale. With the exception of being published in a peer-reviewed journal, no other search limits were applied. The reference list of a previously published review [18] was also screened for relevant studies.

### 2.3 Selection

**Fig. 1:**
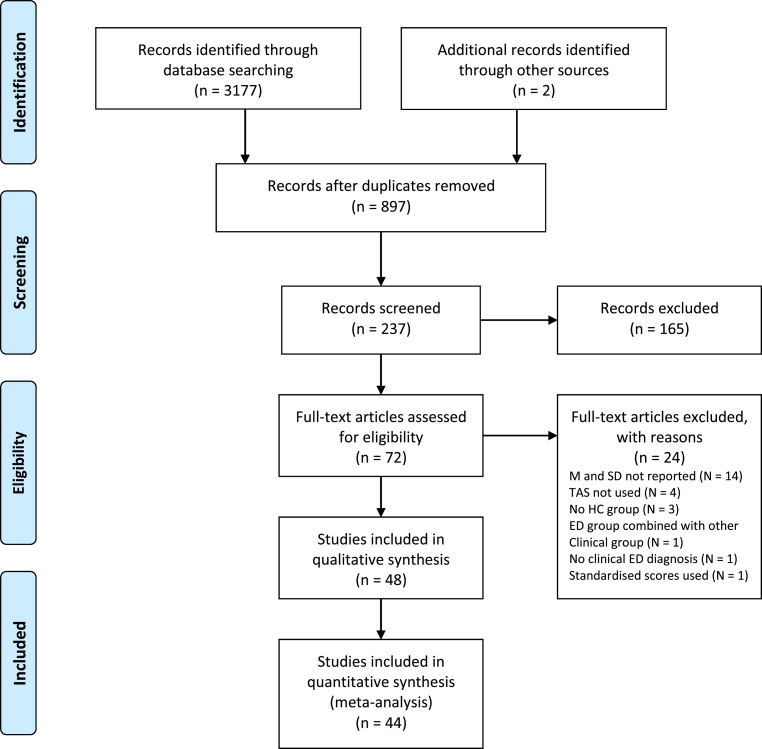
Systematic review search process.

The titles of papers were screened for relevance and the abstracts of those that appeared to meet the criteria were then screened by both the first and second authors. Full texts were retrieved if the abstract indicated that the inclusion criteria had been met or if the details of the study were ambiguous. The first and second author discussed all full-texts and reached consensus about whether to include them in the review. Any full-texts which did not meet the inclusion criteria were excluded. The number of papers reviewed at each stage of the review process, including reasons for exclusion at full-text screening, is displayed in Fig. 1.

### 2.4 Data collection and items

The following data were extracted from each included paper: diagnosis of clinical group; mean age; mean BMI; mean ED duration; how the clinical and HC groups were matched; TAS version; mean TAS scores, including subscale scores if the TAS-20 was used; recruitment site; percentage of female participants; diagnostic tool used and any co-morbidities which were assessed.

### 2.5 Risk of bias in individuals studies

Risk of bias within each study was assessed by considering how the methodology may impact on the results i.e., how clinical groups were matched to HCs, where participants were recruited from and how ED pathology was assessed.

### 2.6 Summary measure

The principle measure used for meta-analysis was the difference in mean scores and standard deviations on the TAS and any reported subscale scores.

### 2.7 Synthesis of data

For the purposes of meta-analyses, studies were split into different ED diagnoses: AN; AN binge-purge type (AN-BP); AN restricting type (AN-R), BN and BED. Studies which included more than one diagnostic group e.g. AN and BN as well as HCs were included in each respective meta-analysis separately. The meta-analyses were performed by pooling the standard effect sizes using a random effects model. This model assumes that as well as within-group variability in scores, mean effect size is also caused by differences between studies. The random-effects model includes between study heterogeneity, resulting in estimates with wider confidence intervals than fixed-effect models. Individual meta-analyses were also run for each of the TAS-20 subscale scores, again split into different diagnostic groups.

### 2.8 Statistical analysis

All meta-analyses were conducted using Review Manager 5.3 [29]. Cohen's *d* [30] was used to estimate effect sizes using the following interpretation: small (0.2), medium (0.5) and large (0.8). Positive effect sizes indicate that the clinical group scored higher on the TAS than the control group. A p value of < 0.05 indicates a significant difference between the clinical group and HCs. To assess the potential impact of moderator variables on the results of the meta-analysis, meta-regression was performed using STATA 13 [31] with the following user-contributed command: *metreg* [32].

### 2.9 Risk of bias across studies

Publication bias was assessed visually by inspection of funnel plots, which represent a plot of a study's precision (1/standard error) against effect size. The absence of studies in the right bottom corner (low precision and small effect sizes) of a funnel plot is usually taken as an indication of publication bias. The Duval and Tweedie [33] non-parametric ‘trim and fill’ method was also used, which accounts for publication bias in meta-analysis as implemented in Stata's user-written command ‘metatrim’ [34]. Effect sizes following adjustment for the publication bias using the trim and fill method are reported. It was noted that the effect size of one study [35] was extremely large, representing an outlier in the AN meta-analysis. The original data were checked and had been reported incorrectly in the paper (standard deviations of the AN and HC groups were incorrect) and have therefore been updated here accordingly.

Between-study heterogeneity was measured using I^2^ [36] based on Cochran's Q test: measure of heterogeneity, I^2^ = 100% × (Q-df)/Q, where df is degrees of freedom. I^2^ ranges between 0%, indicating no heterogeneity and 100%, indicating high heterogeneity with the following approximate interpretation: 0 to 40% might not be important; 30 to 60%, moderate heterogeneity; 50 to 90% may represent substantial heterogeneity and 75 to 100% is considerable heterogeneity [37].

### 2.10 Additional analysis

To examine the potential predictors of between study heterogeneity, age or BMI measures were assessed to examine whether they could explain some of the variance using meta-regressions. Mean age of the clinical and control group and the mean age difference between clinical and control group were used as predictor variables. To assess BMI, BMI of the clinical group and BMI differences between clinical and control group were used. For each domain, three models were assessed: mean age, age difference and mean age and age difference and mean clinical BMI, BMI difference and mean clinical and BMI difference, respectively.

## 3 Results

### 3.1 Study selection

A total of 48 studies were identified through systematic review of the literature. Of these studies, three [24], [38], [39] include a mixed ED sample i.e., patients with either AN, BN or BED and one study [40] included only patients who had recovered from AN. These subgroups of studies were too small to be included in meta-analysis, leaving 44 studies to be included in further analysis. Rommel, Nandrino [41] included an AN-R and a mixed AN-BP/BN group with purging symptoms, therefore, only the AN-R group was included in the corresponding meta-analysis as the mixed group could not be compared to either AN-BP or BN groups in other studies.

### 3.2 Study characteristics

All extracted information included in the systematic review and meta-analyses is presented in Table 1. Generally, the quality of reporting within individual studies was high. All studies included the mean age of participants, aside from Speranza, Corcos [42] who did not report the mean age of HCs. Twelve studies did not report the mean BMI or percentage of ideal body weight (%IBW) for at least one participant group. Half of the studies identified (N = 22) did not report the duration of illness in the clinical ED group. Sixteen studies did not describe how HCs were matched to the clinical group while one study reported the groups being matched by “sociodemographic characteristics” without being more specific [43]. The most common characteristic on which groups were matched was age (N = 29) and 24 studies matched groups on at least two characteristics. Twenty-six studies included at least one group with a sample size of < 30.

Out of a maximum score of 17 on the quality appraisal, three studies scored 16 [44], [45], [46]. The study with the lowest overall quality rating was Deborde, Berthoz [39], which did not include any information on how participants were recruited, inclusion/exclusion criteria, any confounding factors which may have influence the results, the relationship between TAS scores and other variables or the precision or generalisability of the results. All other studies scored between 11 and 15.

Eleven studies used the TAS-26 while the remaining 37 studies used the 20-item version. Of the studies using the TAS-20, 20 reported scores for the three subscales while two [46], [47] included subscale scores for DIF and DDF but not EOT, due to poor internal consistency of this factor within the study. Seven studies did not report where at least one of the groups (clinical or HC) was recruited from and 14 studies did not provide details of how EDs were diagnosed. Co-morbid mental health problems were assessed in all but nine of the included studies, with symptoms of depression and anxiety being the most commonly assessed. Except for Marchesi, Ossola [24], whose ED and HCs samples was 92.3% and 80.8% female respectively and Aloi, Rania [48] whose BED and HC groups were 45% and 81.4% respectively, all studies included only female participants. Tchanturia, Davies [49] did not explicitly report the sex of their participants.

Twenty-two studies attempted to control for potential confounding variables within analysis, 19 of which controlled for depression. After controlling for depression, the difference in TAS scores between the clinical group and HCs remained significant in eight studies. In the other studies, the difference was either no longer significant or was only significant on certain subscales of the TAS or for subgroups of participants. Thirty-four studies also examined correlations between TAS scores and other variables, including depression, anxiety, BMI and illness duration, of which 15 studies reported a significant positively correlation between TAS scores and depression in their respective clinical groups. More information on the results of the quality appraisal are presented in the appendix.

### 3.3 Risk of bias

**Fig. 2:**
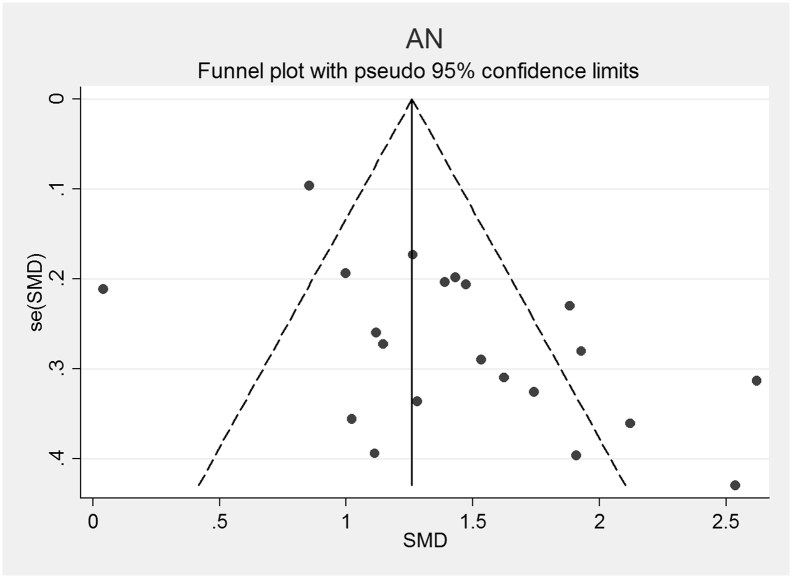
Funnel plot of Anorexia Nervosa (AN) studies included in the meta-analysis to assess for publication bias. Each dot represents a study included in the meta-analysis, with the Y axis representing the size of each study and the X axis, each study's result.

**Fig. 3:**
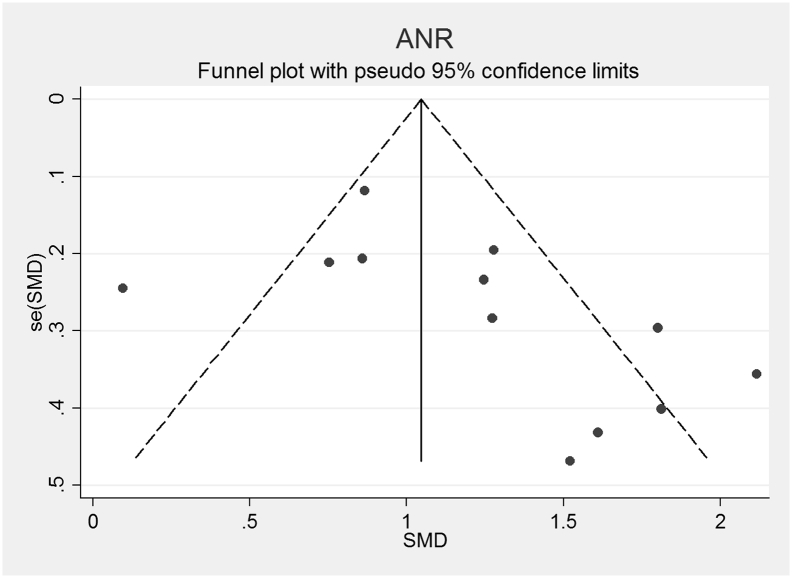
Funnel plot of Anorexia Nervosa, Restricting subtype (AN-R) studies included in the meta-analysis to assess for publication bias. Each dot represents a study included in the meta-analysis, with the Y axis representing the size of each study and the X axis, each study's result.

**Fig. 4:**
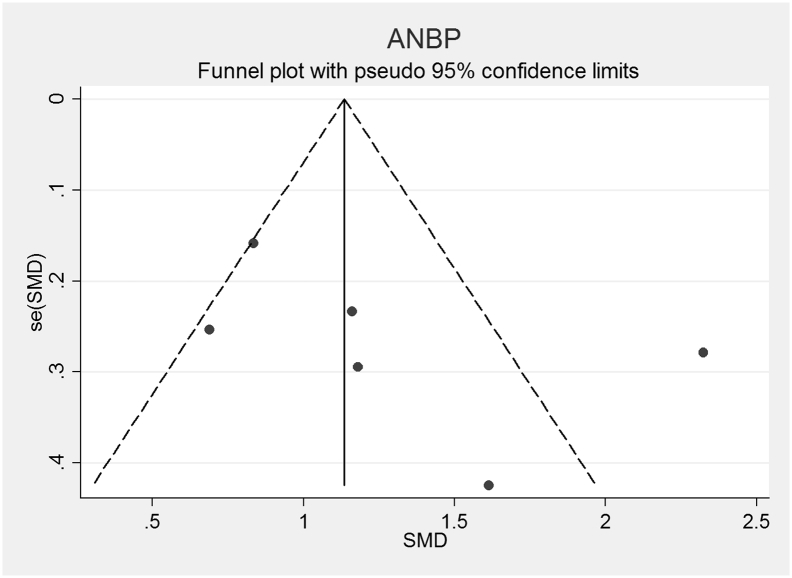
Funnel plot of Anorexia Nervosa, Binge-Purge subtype (AN-BP) studies included in the meta-analysis to assess for publication bias. Each dot represents a study included in the meta-analysis, with the Y axis representing the size of each study and the X axis, each study's result.

**Fig. 5:**
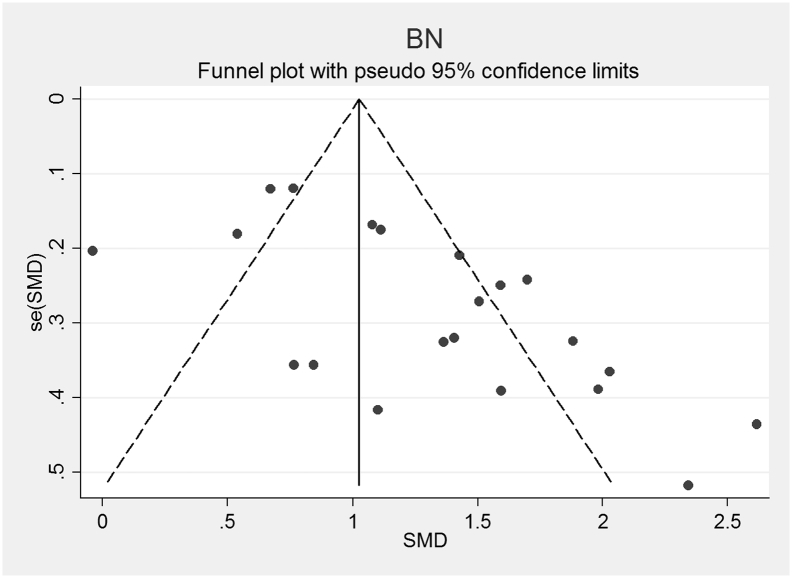
Funnel plot of Bulimia Nervosa (BN) studies included in the meta-analysis to assess for publication bias. Each dot represents a study included in the meta-analysis, with the Y axis representing the size of each study and the X axis, each study's result.

**Fig. 6:**
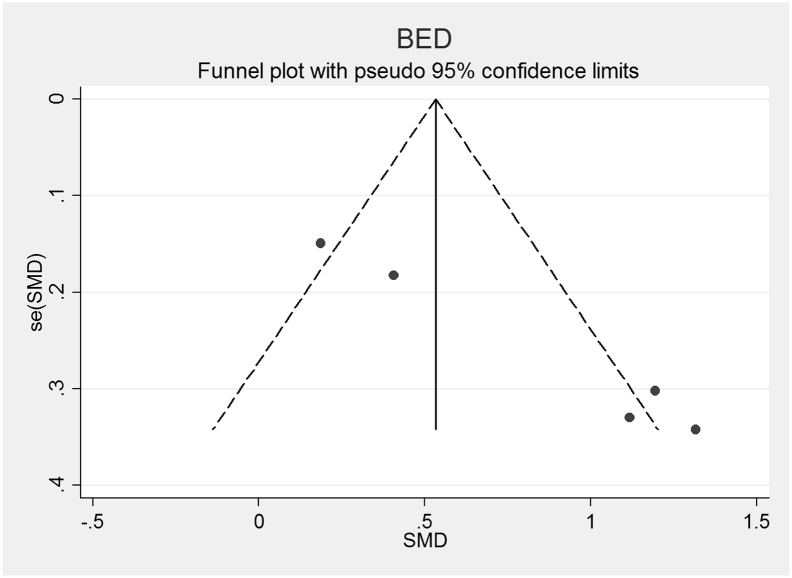
Funnel plot of Binge Eating Disorder (BED) studies included in the meta-analysis to assess for publication bias. Each dot represents a study included in the meta-analysis, with the Y axis representing the size of each study and the X axis, each study's result.

The funnel plots for AN, AN-R, AN-BP, BN and BED studies are shown in Fig. 2, Fig. 3, Fig. 4, Fig. 5, Fig. 6. In all five study groups, there was some evidence of publication bias as there was a small asymmetrical appearance of the funnel plots with a gap in the left bottom corner of the graph, indicating that smaller effect sized studies with less precision may be missing. The trim and fill method indicated missing studies in all five groups. The re-estimated effect sizes decreased but remained significant (p < 0.001) or almost significant (BED: p = 0.065), (AN: 1.08 (95% CI 0.83, 1.33), AN-R: 10.94 (95% CI 0.65, 1.24), AN-BP: 1.89 (95% CI 0.39, 1.1.39), BN: 0.90 (95% CI 0.64, 1.16), BED: 0.44 (95% CI − 0.3, 0.92, p = 0.065)).

### 3.4 Synthesis of results

**Fig. 7:**
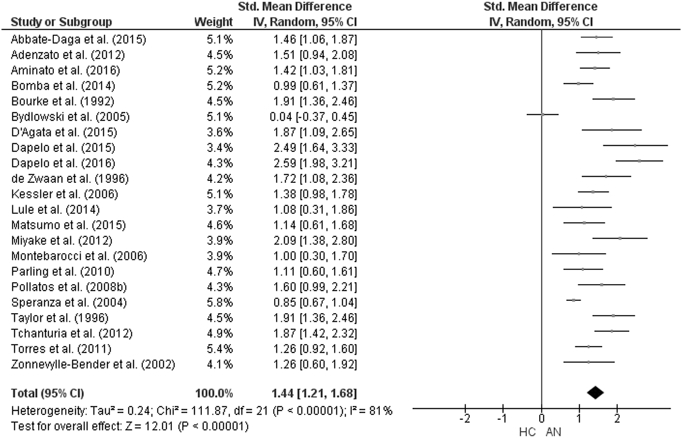
Forest plot of mean TAS score: standardized mean effect size for differences (SMD) between Anorexia Nervosa (AN) and Healthy Controls. CI, confidence interval.

**Fig. 8:**
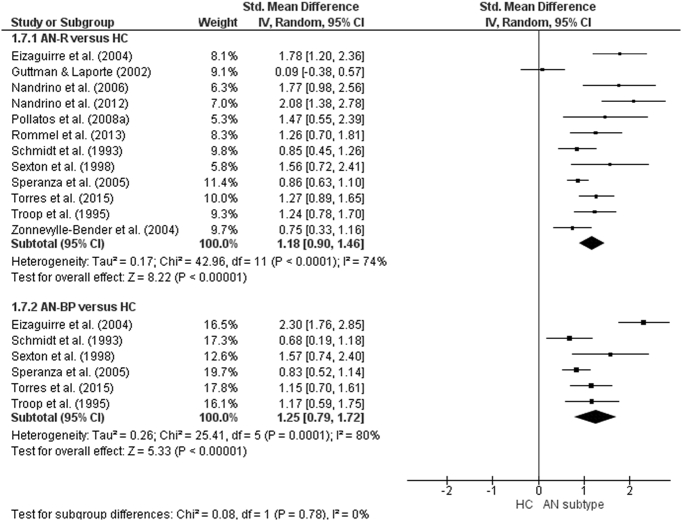
Forest plot of mean TAS score: standardized mean effect size for differences (SMD) between Anorexia Nervosa, Restricting subtype (AN-R) and Healthy Controls (HC) and between Anorexia Nervosa, Binge-Purge subtype (AN-BP) and HCs. CI, confidence interval.

**Fig. 9:**
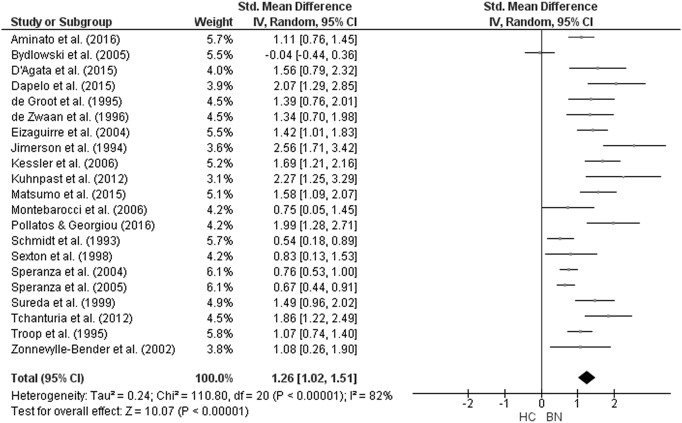
Forest plot of mean TAS score: standardized mean effect size for differences (SMD) between Bulimia Nervosa (BN) and Healthy Controls. CI, confidence interval.

**Fig. 10:**
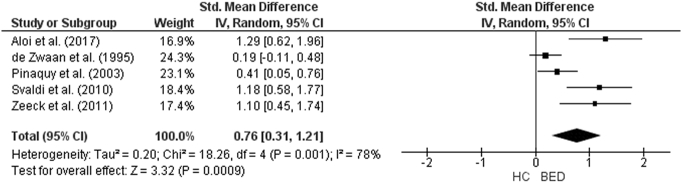
Forest plot of mean TAS score: standardized mean effect size for differences (SMD) between Binge Eating Disorder (BED) and Healthy Controls. CI, confidence interval.

The forest plot of studies including participants with AN is displayed in Fig. 7. The random-effect analysis with a total sample size of 2332 participants (AN = 944, HC = 1388) from 22 studies revealed a significant difference with a large effect size between AN and HC groups on the total TAS score (*d* = 1.44, (95% CI 1.2, 1.68) z = 12.01, p < 0.001). For studies in which AN subtype was defined, the forest plots are displayed in Fig. 8. For AN-R studies, the analysis with a total sample size of 1159 participants (AN-R = 441, HC = 718) from 12 studies revealed a significant difference between groups, again with a large effect size (*d* = 1.18, (95% CI 0.90, 1.46) z = 8.22, p < 0.001). For AN-BP studies, the analysis with a total sample size of 720 participants (AN-BP = 177, HC = 543) from six studies revealed a significant difference with large effect size (*d* = 1.25, (95% CI 0.79, 1.72) z = 5.33, p < 0.001). The forest plot of BN studies is displayed in Fig. 9. The analysis included a total of 2391 participants (BN = 858, HC = 1533) from 21 studies and revealed a significant difference with a large effect size between the two groups (*d* = 1.26, (95% CI 1.02, 1.51) z = 10.07, p < 0.001). The forest plot for BED studies is displayed in Fig. 10. The analysis included a total of 488 participants (BED = 192, HC = 296) from five studies and revealed a significant differences with a medium effect size between the two groups (*d* = 0.76, (95% CI 0.31, 1.21) z = 3.32, p < 0.001).

### 3.5 TAS-20 subscale analysis

**Fig. 11:**
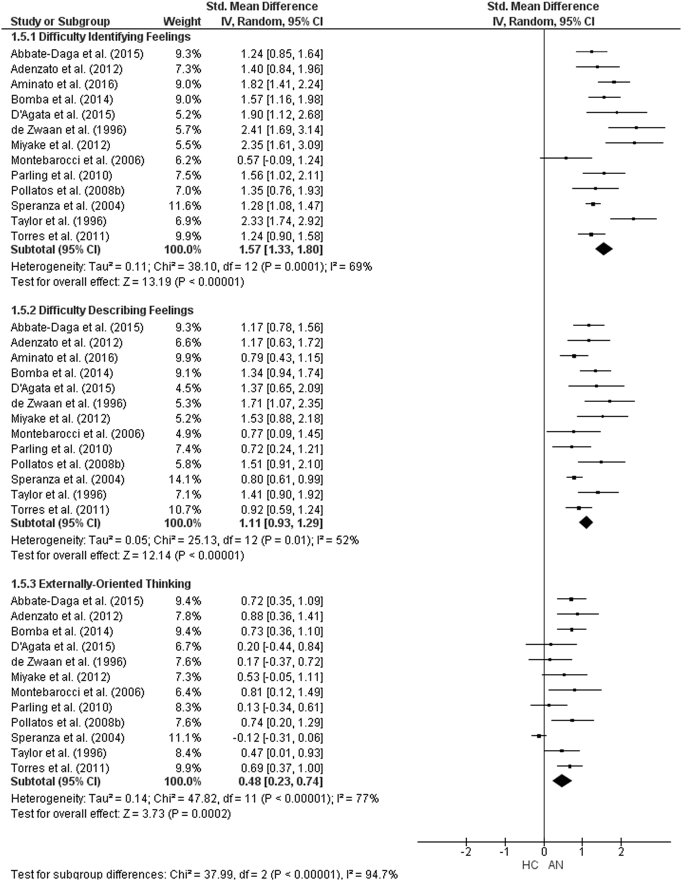
Forest plot of mean TAS subscale scores: standardized mean effect size for differences (SMD) between Anorexia Nervosa (AN) and Healthy Controls (HC). CI, confidence interval.

**Fig. 12:**
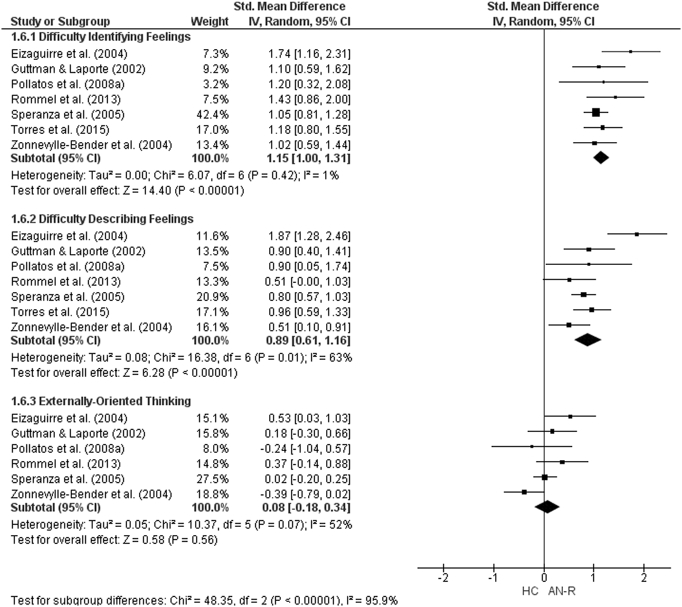
Forest plot of mean TAS subscale scores: standardized mean effect size for differences (SMD) between Anorexia Nervosa, Restricting subtype (AN-R) and Healthy Controls (HC). CI, confidence interval.

**Fig. 13:**
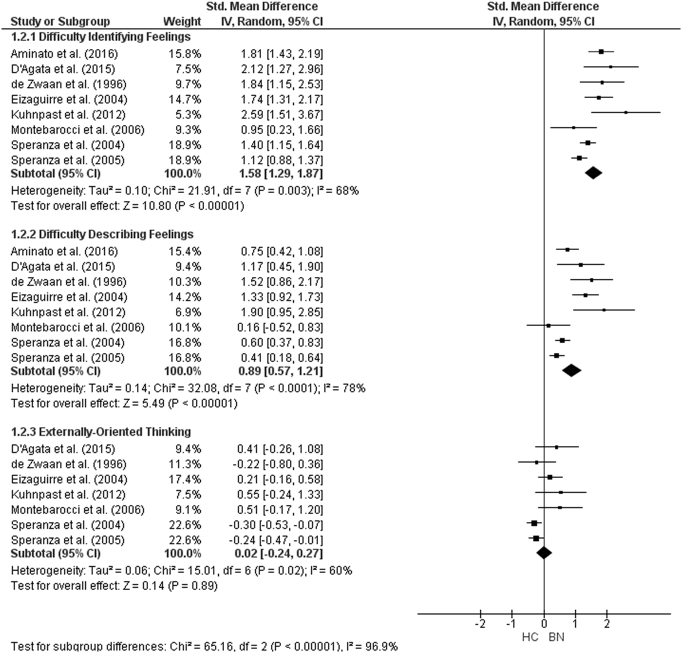
Forest plot of mean TAS subscale scores: standardized mean effect size for differences (SMD) between Bulimia Nervosa (BN) and Healthy Controls (HC). CI, confidence interval.

The forest plots for TAS-20 subscale analysis are shown in Fig. 11, Fig. 12, Fig. 13. For AN studies, the random-effect analysis included a total sample size of 1642 (AN = 635, HC = 1007) from 13 studies for the DIF and DDF subscales. For the DIF subscale, there was a significant difference between groups with a large effect size (*d* = 1.57, (95% CI 1.33, 1.80) z = 13.1933, p < 0.001). For the DDF subscale, there was also a significant difference between groups with a large effect size (*d* = 1.11, (95% CI 0.93, 1.29) z = 12.14, p < 0.001). For the EOT subscale, the analysis included 1509 participants (AN = 582, HC = 927) from 12 studies. For the EOT subscale, there was a significant difference between groups with a small effect size (*d* = 0.48, (95% CI 0.23, 0.74) z = 3.73, p < 0.001).

For AN-R studies, the meta-analysis of the DIF and DDF subscales included a total of 833 participants (AN-R = 301, HC = 532). As was the case in the AN studies, there were significant differences between the groups on the DIF (*d* = 1.15, (95% CI 1.00, 1.31), z = 14.4, p < 0.001) and DDF (*d* = 0.89, (95% CI 0.61, 1.16) subscales with large effect sizes. The analysis for the EOT subscale, consisting of a total sample size of 701 (AN-*R* = 249, HC = 452) revealed that there was no significant difference between groups (*d* = 0.08, (95% CI -0.18, 0.34) z = 0.58, p = 0.56). As only three studies including participant with AN-BP reported TAS-20 subscale scores, meta-analysis could not be run for this diagnostic group.

For BN, the analysis for the DIF and DDF subscales consisted of a total sample size of 1400 (BN = 400, HC = 1000) from eight studies., There were significant differences with large effect size between groups on both the DIF (*d* = 1.58, (95% CI 1.29, 1.87) z = 10.80, p < 0.001) and the DDF (*d* = 0.89, (95% CI 0.57, 1.21) z = 5.49, p < 0.001) subscale scores. There was no difference between the groups on the EOT subscale score (*d* = 0.02 (95% CI − 0.24, 0.27) z = 0.14, p = 0.89).

### 3.6 Additional analysis

There was evidence of substantial heterogeneity in the overall TAS score analysis for all diagnostic groups (AN, I^2^ = 81%; AN-R, I^2^ = 74%, AN-BP, I^2^ = 80%; BN, I^2^ = 82%, BED, I^2^ = 78%). To examine the potential cause of this heterogeneity, meta-regression was performed with BMI of the clinical group, differences in BMI between clinical and control group, mean age and age difference between clinical and control group as moderator variables. Meta regressions revealed that BMI of the clinical groups or the difference in BMI between clinical and control group revealed that only in the AN group there was a significant positive effect of BMI difference on effect size (b = 0.21 (95% CI 0.03, 0.38), t = 2.56, p = 0.023) However, the effect remained significant at the mean difference (*d* = 1.10 (95% CI 0.66, 1.53), t = 5.43, p < 0.001 (all other p's > 0.1). There was a significant effect of mean age on outcome in AN and BN (AN: b = 0.10 (95% CI 0.05, 0.16), t = 3.91, p = 0.001, N = 22; BN: b = 0.09 (95% CI 0.003, 0.18), t = 2.19, p = 0.042, N = 21) but not for age difference between clinical and control groups (AN: b = 0.02 (95% CI − 0.20, 0.23), t = 0.18, p = 0.86, N = 21; BN: 0.002 (95% CI − 0.10, 0.11), t = 0.05, p = 0.96, N = 20). The effect of age was usually (not in BED) smaller and not significant in AN-R (b = 0.01 (95% CI − 0.34, 0.36), t = 0.06, p = 0.96, N = 6) or AN-BP (b = 0.05 (95% CI − 0.07, 0.18), t = 0.93, p = 0.38, N = 12) or BED b = 0.17(− 0.1–0.44) t = 2.06, p = 0.13 and age difference was not significant: AN-R: b = − 0.09 (95% CI − 0.37, 0.18), t = − 0.93, p = 0.40, N = 6; AN-BP: b = − 0.04 (95% CI − 0.17, 0.10), t = − 0.59, p = 0.57, N = 12 or BED b = − 0.01 (95% CI − 24, − 0.21), t = − 0.19, p = 0.86, *N* = 5. Conclusions did not change if both variables were modelled together in a multiple regression (AN: age: b = 0.11 (95% CI 0.05, 0.17), t = 4.13, p = 0.001, age difference: b = − 0.10 (95%. CI − 0.28, 0.07), t = − 1.28, p = 0.22, N = 21, BN: age: b = 0.09 (95% CI 0.01, 0.19), t = 2.13, p = 0.048, age difference: b = − 0.02 (95%. CI − 0.12, 0.08), t = − 0.51, p = 0.62, N = 20).

## 4 Discussion

The aim of this review was to synthesise the literature on alexithymia in EDs using the TAS, a widely used self-report measure. A total of 48 studies were identified through systematic review, 44 of which were included in meta-analyses examining group differences in total TAS scores. Twenty-two AN studies, 12 AN-R studies, six AN-BP studies, 21 BN studies and five BED studies were included in the meta-analyses. There were significant differences between all diagnostic groups and HCs on the total TAS score with large effect sizes, with the exception of BED where the difference was with a medium effect size. This indicates that individuals across the spectrum of EDs have more difficulties with identifying and describing their emotions than individuals without EDs. When the individual subscale scores of the TAS-20 were analysed, individuals with AN scored significantly higher on all subscales than HCs. However, in studies where the AN subtype was defined as AN-R and in BN studies, the clinical group only scored higher on the Difficulty Identifying Feelings and Difficulty Describing Feelings subscales. Due to lack of data, it was not possible to examine subscale scores in individuals with AN-BP or BED.

There was evidence of publication bias in all diagnostic groups. This may have been accounted for by the exclusion of any studies that did not report both the mean and standard deviations of TAS scores. Further attempt to contact the authors of such papers may have therefore been beneficial. In addition, methodological bias may have impacted on the results. For example, a large proportion of studies had a relatively small sample size, with very few accounting for this with a power size calculation. When adjusting for publication bias, the effect sizes remained significant, however, for BED the significance disappeared. Further studies examining alexithymia in individuals with BED are therefore needed to confirm whether they have real difficulties with identifying and labelling emotions.

There were high levels of heterogeneity (74–82%) across studies. To examine potential reasons for this heterogeneity, BMI of the clinical group, differences in BMI between groups, age and age differences between groups were entered as moderator variables into meta-regression. The BMI of the clinical groups did not influence the effect size for any of the ED diagnoses. In the AN analysis, there was a significant effect of BMI difference between the clinical and HC group on effect size. There was also a significant effect of mean age of the clinical groups on outcome in AN and BN studies, with older age of the clinical group being associated with a larger effect size.

The acute phase of AN is associated with reduced facial expression of emotions compared with recovered patients [85], suggesting that starvation may impact on alexithymia scores. As older patients may be expected to have longer illness durations than younger patients, this could explain why age was associated with a larger effect size. It is also possible that older patients experience more co-morbidities, including Obsessive-Compulsive Disorder and depression, which are also associated with alexithymia [2]. In the general population, alexithymia has also been associated with increasing age [86]. Other reasons for the large heterogeneity between studies include factors such as the level of comorbid psychiatric conditions, such as anxiety or depression, which could not be controlled for in meta-regression. There is also some evidence that treatment outcome, or indeed the treatment which patients receive, may be associated with levels of alexithymia in ED patients [87] and thus the type of treatment patients in each of the studies were receiving may have also accounted for some of the heterogeneity observed.

Despite the large number of cross-sectional studies examining alexithymia in EDs, longitudinal studies examining changes in TAS scores overtime are lacking. One study [88] examined the predictive value of alexithymia over three-years in patients with EDs. Using the TAS-20, the DIF factor was a significant predictor of treatment outcome, independent of both depression and eating disorder severity. In addition, there was significant improvement in alexithymia scores, along with improvement in clinical severity and depression, suggesting that alexithymia may not be stable in individuals with EDs. The one study identified in this review which included recovered patients [40] found no significant difference on TAS-20 scores between the clinical and HC groups. This suggests that starvation, or the acute phase of AN may impact on alexithymia. However, as illness duration was not included in meta-regression, it is not possible to draw conclusions about its impact on alexithymia in individuals with EDs.

Interestingly, while BMI of the clinical groups did not influence the effect size, in AN studies, the difference in BMI between the two groups did have a significant positive effect on effect size, with a greater difference in BMI being associated with a larger effect size. This suggests that BMI may be associated with alexithymia in AN although after controlling for this, the main effect was still significant. Three studies included in the AN meta-analysis [47], [50], [53] controlled for BMI in their analysis and found that group differences remained significant. There is also a possibility that this is a chance result which would disappear after controlling for multiple testing. One previous study [89] found that lower BMI was associated with decreased difficulties with emotion regulation in women with acute AN. While emotion regulation is a separate construct to alexithymia, with the former referring to the ability to respond appropriately to situations with a range of emotions, one might expect the two to be linked. Further research may therefore be warranted to explore the impact of age, BMI and illness duration on both alexithymia and emotional regulation.

Around half of the studies attempted to control for the effect of potentially confounding variables within the analysis of group differences in TAS scores. Of the 19 studies which controlled for depression, the differences in TAS scores between clinical and HC groups only remained significant in eight. In the eleven studies whose results became insignificant after controlling for depression, eight were conducted with individuals with AN (including AN-R and AN-BP). Six studies [24], [43], [44], [45], [54], [67] also controlled for anxiety within the analysis, however, so it is not possible to determine whether group differences were influenced by depression, anxiety or both. Given the previous literature suggesting a link between alexithymia and depression [e.g., 9], research aiming to elucidate the relationship between the two constructs i.e., with inclusion of a clinical control group with depression, would be beneficial.

These meta-analyses indicate that difficulties with identifying and describing emotions are transdiagnostic across the spectrum of EDs. This is consistent with a previous systematic review of alexithymia [18] and extends previous research by demonstrating that the differences between clinical groups and HCs are of the same magnitude i.e., large effect sizes, across ED diagnoses. Nowakowski, McFarlane [18] found that individuals with EDs score higher on the DIF and DDF subscales of the TAS-20 but not on the EOT subscale. In this meta-analysis, individuals with AN were found to score significantly higher on all sub-scales, including the EOT whereas in AN-R studies and BN studies there was no difference between groups on the EOT subscale. This suggests that difficulties with EOT may be diagnosis-specific although further research to confirm this is warranted. Cronbach's alpha has been shown to be lower for EOT than for the other two factors and both DDF and DIF have low correlations with EOT, possibly due to low internal consistency of this factor [90]. This may also explain the reason for the inconsistent findings across EDs on the EOT subscale in these meta-analyses.

Compared with other ED diagnoses, only five studies using the TAS were identified in individuals with BED and it was not possible to conduct subscale analysis on these studies, due to only two of them [48], [70] including subscale scores. Future research is therefore needed to determine with difficulties across the TAS subscales are present in BED. In addition, studies examining the presence of alexithymia in individuals who have recovered from an ED will help delineate the relationship between alexithymia and ED psychopathology.

Despite individuals with EDs scoring significantly higher than HCs, it is still not clear whether the TAS is measuring a universal construct of alexithymia or whether it is instead measuring other traits such as negative affect, emotional expressivity or social shame [22], [23], [24]. Differences in effect size on the TAS-20 subscale scores indicate that the TAS may be measuring several different constructs and suggests that the proposed three-factor model may not be suitable for use with ED populations [25]. Self-report measures such as the TAS may not be reliable in that the very nature of alexithymia may make it difficult for individuals to reflect on their emotions, thus giving inaccurate report. For this reason, the development of tools which accurately assess the nature of difficulties with emotion recognition using more objective measures would be beneficial. This would ensure that co-morbidities such as anxiety or depression can be adequately controlled for in the assessment of alexithymia and would allow for meaningful comparisons between clinical groups.

The Observer Alexithymia Scale [OAS; 91], an informant-report measure, was developed as an alternative way of measuring alexithymia and can be completed by either relatives or clinicians. In a study examining the use of the OAS in an eating disorder population, the measure showed acceptable validity and inter-rater reliability and the OAS was recommended for use alongside the TAS-20 in both research and clinical practice [92]. The psychometric properties of the OAS have been tested across a range of psychiatric disorders and it was found to be psychometrically sound for evaluating observer ratings of alexithymia [93]. Despite this, correlation between the TAS-20 and OAS is reportedly weak [94] and the authors do not recommend its use in clinical settings. Thus, disagreement exists over exactly which constructs the OAS and TAS are measuring. Another informant measure, the Toronto Structured Interview for Alexithymia [TSIA; 95] allows for multi-modal assessment of alexithymia and was designed as a tool for clinicians to elicit information about the extent of a patient's difficulties with DIF, DDF, EOT and fantasy and imaginal processes. When used with females with AN and their parents, there was significant discordance between the two measures, with suggestion that the TSIA may be more sensitive for detecting alexithymia than the self-report TAS-20 [96]. Further studies using the TSIA or other clinical-led assessments across psychiatric populations would therefore help determine whether such tools were measuring independent, related or homogenous constructs.

The findings from the current meta-analysis add to the wider literature on socio-emotional difficulties in EDs. For example, a review [97] used a multidimensional framework to map out emotion regulation difficulties in AN and BN. The model [98] outlines four dimensions theorised to contribute to the development or maintenance of psychopathology: use of adaptive and situationally appropriate emotion regulation strategies; impulse inhibition and behavioural control when distressed; emotional awareness, clarity, and acceptance; and emotional approach and tolerance. There is evidence to suggest those with AN and BN have difficulties across all four dimensions, however most relevant here are difficulties with emotional awareness and acceptance, including several constructs which overlap with alexithymia. For example, using the Levels of Emotional Awareness Scale [LEAS; 99], which asks individuals to report how they and another person would feel in various scenarios, several studies have found impairments in emotional awareness in the self and others in both EDs [44], [100]. Studies using experimental paradigms such as the LEAS while controlling for levels of alexithymia would help elucidate the relationships between different socio-emotional constructs in people with EDs.

Relatedly, emotional theory of mind, the ability to infer the emotional states of others, is also reported to be impaired in AN and BN [101], [102], but may improve with recovery [100]. Facial emotion recognition also appears to be impaired in AN (but not BN), although results vary somewhat for different emotions [103], [104]. Finally, there is evidence from both self-report and performance-based measures for greater emotional suppression and non-acceptance in AN and BN compared to controls [105], [106], [107]. Interestingly, this emotional suppression appears to also be reflected in facial emotion expressivity, with individuals with AN showing significantly less positive emotions than HCs, and BN showing an intermediate profile [108]. Given the difficulties in recognising ones' own emotions, it is perhaps not surprising that those with EDs show widespread problems in decoding the emotional states of others.

Given the difficulties that individuals with EDs have identifying and describing emotions, clinical intervention has recently shifted focus on addressing these issues. Certain treatment protocols including Cognitive Remediation and Emotion Skills Training (CREST) have attempted to address these difficulties in both individual and group format [109], [110], with preliminary findings suggesting that such treatment leads to improvements in patients' ability to label emotions and a reduction in social anhedonia. The development of treatments such as CREST are in their infancy and thus further studies assessing their efficacy in reducing alexithymia are needed. Other treatment modalities such as the Maudsley Model of Anorexia Nervosa Treatment in Adults (MANTRA; Schmidt et al., 2015) and Radically Open Dialectical Behavioural Therapy (RO-DBT; Lynch et al., 2013) also include a focus on emotional difficulties. Another relatively new treatment for EDs, namely Emotion Acceptance Behaviour Therapy [EABT; 111], aims to combine standard behavioural therapy with strategies to increase emotional awareness and has been associated with decreased emotion avoidance at follow-up. Measuring alexithymia before and after the treatment will be beneficial to explore the effectiveness of these approaches on improving emotion recognition.

### 4.1 Limitations

The studies varied greatly in terms of the mean age of participants, mean BMI, mean illness duration of the clinical group, matching criteria to HCs, recruitment sites, diagnostic tools and co-morbidities assessed. This heterogeneity made direct comparison between studies difficult. Only six studies [46], [50], [52], [80], [112], [113] fully reported all data extracted for the purpose of this review. The variety of sociodemographic characteristics, recruitment sites and diagnostic tools may have accounted for some of the between-study heterogeneity found within the analysis, although this could not be accounted for within analysis. Analysis of risk of bias across studies indicated that smaller effect sized studies with less precision may be missing. There is a possibility that a small number of studies were not identified through systematic review due to not being published in English or full texts being unavailable.

## 5 Conclusion

Alexithymia, particularly difficulties with identifying and describing emotions, is transdiagnostic across the ED spectrum. Our systematic review of the literature, focusing on the TAS demonstrated that individuals with AN, BN and BED score consistently higher on the TAS than HCs. Despite this, current instruments which measure alexithymia may be influenced by co-morbid symptoms such as depression or anxiety and may not be measuring a homogenous construct. Recognising and managing emotions are viable treatment targets. Future research should focus on improving the measurement of this construct and on the development of effective clinical intervention to address difficulties with emotional recognition.

## Role of funding sources

This research did not receive any specific grant from funding agencies in the public, commercial, or not-for-profit sectors.

## Contributors

The first and fourth authors conceived and designed the study. The first and second authors conducted the systematic review and data extraction. The first and third authors conducted the data-analysis. The first author prepared the first draft of the manuscript. All authors contributed to and approved the final manuscript.

## Conflict of interest

There are no known conflicts of interest associated with this publication and there has been no significant financial support for this work that could have influenced its outcome. We confirm that the manuscript has been read and approved by all named authors and that there are no other persons who satisfied the criteria for authorship but are not listed.

We further confirm that the order of authors listed in the manuscript has been approved by all of us. We confirm that we have given due consideration to the protection of intellectual property associated with this work and that there are no impediments to publication, including the timing of publication, with respect to intellectual property. In so doing we confirm that we have followed the regulations of our institutions concerning intellectual property.

We understand that the Corresponding Author is the sole contact for the Editorial process (including Editorial Manager and direct communications with the office). He/she is responsible for communicating with the other authors about progress, submissions of revisions and final approval of proofs. We confirm that we have provided a current, correct email address which is accessible by the Corresponding Author and which has been configured to accept email from.

## Appendix A Supplementary data

Supplementary material Quality appraisal of studies included in systematic review. Image 1
