# Supplementary material for: Alexithymia in eating disorders: Systematic review and meta-analyses of studies using the Toronto Alexithymia Scale
Source: J Psychosom Res. 2017 Aug;99:66–81. doi: 10.1016/j.jpsychores.2017.06.007 (PMC5986724; doi:10.1016/j.jpsychores.2017.06.007)
Supplement: Supplementary material — Quality appraisal of studies included in systematic review. [file mmc1.docx]

| **Study** | **Clearly focused issue?** | **Appropriate method?** | **Case recruitment** | | | | **Control recruitment** | | | | **Exposure accurately measured** | | **Confouding factors accounted for in analysis? If so, what were the results?** | **Correlation between other variables e.g., BMI and alexithymia assessed? If so, what was the result?** | **Precision of results** | **Believe results?** | **Results applicable?** | **Do results fit with available evidence?** | **Score (max 17)** |
| --- | --- | --- | --- | --- | --- | --- | --- | --- | --- | --- | --- | --- | --- | --- | --- | --- | --- | --- | --- |
|  |  |  | **Cases defined?** | **Recruitment source given?** | **Exclusion/inclusion criteria?** | **Power calculation?/ sample size?** | **Controls defined?** | **Recruitment?** | **Exclusion/ inclusion?** | **Matched to cases?** | **TAS described?** | **Procedure?** |  |  |  |  |  |  |  |
| Abbate-Daga et al. (43) | Y | Y | Y | Y | Y | N | Y | Y | Y | Sex | Y | N | After controlling for anxiety, depression, BMI & illness duration (for AN), correlations between TAS-DIF and TAS-TOT and intolerance of uncertainty remained significant. | AN - Alexithymia negatively correlated with duration of illness, and positively correlated with stait & trait anxiety, and depression. HC - alexithymia positively correlated with BMI, stait & trait anxiety, and depression. | Y | Y | Y | Y | 15 |
| Adenzato et al. (44) | Y | Y | Y | Y | Y | N | Y | N | Y | Age, education, sex | Y | Y | After controlling for BMI, depression, age of onset, and illness duration (AN group) the correlation between perceived social support and TAS-20 remained significant. | AN group only - No correlation between BMI, age at illness onset, or illness duration and any of the experimental or clinical tasks. The TAS-20 positively correlated with depression, and negatively correlated with perceived social support and the empathy quotient. | Y | Y | Y | Y | 15 |
| Aloi et al. | Y | Y | Y | Y | Y | N | Y | Y | Y | N | Y | Y | After adjusting for gender, depression, and alexithymia, differences between groups in EDI-2 subscale remained significant, except for ineffectiveness and impulse regulation. The significant group effect on TAS-20 scores remained significant after controlling for gender and depression. | TAS-20 scores positively correlated with depression, binge eating scale, and interoceptive awareness, and negatively correlated with empathy quotient and facial emotion recognition. | Y | Y | Y | Y | 15 |
| Amianto et al. | Y | Y | Y | Y | Y | N | Y | Y | N | Age, sex | Y | Y | After correcting for age and BMI, group differences in TAS-20 scores remained significant. | TAS-20 scores negatively correlated with self-directedness and interoceptive awareness. | Y | Y | Y | Y | 15 |
| Bomba et al. (45) | Y | Y | Y | Y | Y | N | Y | Y | Y | Age, sex | Y | Y | N | N | Y | Y | Y | Y | 14 |
| Bourke et al. (46) | Y | Y | Y | Y | N | N | Y | Y | N | Age, education, sex | N | Y | Splitting the AN group into various dichotomous groups, there were no differences in TAS scores between the following: those who had been ill <2yrs vs. those who had been ill >2yrs; patients who were >20% of expected weight vs. patients who were <20% of expected weight; and those scoring above cut off on the CCEI depression subscale vs. those scoring below. | TAS scores negatively correlated with education in the AN group. | Y | Y | Y | Y | 13 |
| Bydlowski et al. (47) | Y | Y | Y | Y | N | Y | Y | Y | Y | Age, education, SES, sex | Y | Y | Significant differences in TAS scores between groups remained significant after controlling for age, education, & medication. However, after controlling for depression, patients TAS scores were not significantly different from controls. Conversely, after controlling for anxiety, group differences in TAS scores remained significant. | In both groups, there was a trend toward TAS and Levels of Emotional Awareness Scale (LEAS) scores to be negatively correlated. TAS scores were positively correlated with depression and anxiety in both groups. There was no correlation between TAS score and duration of illness. | Y | Y | Y | Y | 16 |
| D'Agata et al. (48) | Y | Y | Y | Y | Y | N | Y | Y | Y | Sex | Y | N | Controlling for age, anxiety, and depression, differences between groups in TAS-20 total scores became non-significant, however differences in scores on the difficulty describing and identifying feelings sub-scales remained significant. | TAS-20 total scores were significantly positively correlated with anxiety in BN, and with depression in AN. | Y | Y | N (? TOO SMALL SAMPLE SIZE?) | Y | 14 |
| Dapelo et al. (32) | Y | Y | Y | Y | Y | Y | Y | Y | Y | Sex | Y | Y | N | TAS-20 scores were negatively associated with non-duchene smile duration. | Y | Y | N (? TOO SMALL SAMPLE SIZE?) | Y | 15 |
| Dapelo et al. (49) | Y | Y | Y | Y | Y | N | Y | Y | Y | Age, education, sex | Y | Y | N | N | Y | Y | Y | Y | 14 |
| de Groot et al. (50) | Y | Y | Y | Y | N | N | Y | Y | N | Sex | Y | Y | When depression was controlled for, a trend remained for the BN group to have higher TAS scores than HCs. | TAS scores were significantly positively correlated with depression in BN (at both admission and discharge) and HCs. TAS scores at discharge were significantly positively correlated with purge frequency in BN. | Y | Y | Y | Y | 14 |
| De Panfilis et al. (34) | Y | Y | Y | Y | Y | N | Y | Y | Y | Sex | Y | Y | N | N | Y | Y | Y | Y | 14 |
| de Zwaan et al. (51) | Y | Y | Y | Y | Y | N | Y | N | Y | Sex | Y | Y | When depression was controlled for, differences between groups' TAS-20 scores remained significant. | N | Y | Y | Y | Y | 14 |
| de Zwaan et al. (37) | Y | Y | Y | Y | Y | N | Y | Y | Y | Sex | Y | Y | High scores on EDI subscales (interpersonal distrust and ineffectiveness) as well as low educational level were independent predictors of TAS scores (explaining 27.3% of variance). | N | Y | Y | Y | N | 15 |
| Deborde et al. (35) | Y | Y | Y | N | N | N | Y | N | N | N | Y | Y | N | N | N | Y | N (TOO SMALL ED SAMPLE?) | Y | 7 |
| Eizaguirre et al. (42) | Y | Y | Y | Y | Y | N | Y | N | Y | Sociodemographic characteristics | Y | Y | When controlling for anxiety and depression, the relationship between Eating Attitude Test (EAT) and TAS-20 scores disappeared. When controlling for depression and anxiety co-ordinately, there were no significant differences among groups on the TAS-20. | TAS-20 scores positively correlated with depression and anxiety in ED and HCs. In ED, TAS-20 scores were positively correlated with EAT scores, and negatively correlated with BMI, | Y | Y | Y | Y | 15 |
| Guttman & Laporte (52) | Y | Y | Y | Y | Y | N | Y | Y | Y | Sex | Y | Y | N | Those who scored above cut-off on the TAS had significantly less empathy and more personal distress than those scoring below cut-off. They also had higher scores on the SCL-90-R. | Y | Y | Y | Y | 15 |
| Jimerson et al. (53) | Y | Y | Y | Y | Y | N | Y | Y | Y | Age, weight, sex | Y | Y | When controlling for anxiety and depression, differences between HCs and BN on TAS total and TAS F1 (but not F2) remained significant. | TAS scores were positively correlated with SSPS-R scores, depresssion, and anxiety | Y | Y | N (Too small sample?) | Y | 15 |
| Kessler et al. (54) | Y | Y | Y | Y | N | N | Y | Y | Y | Age, sex | Y | Y | N | No significant correlations between facial emotion recognition, TAS scores, ED symptoms, and SCL-90-R scores. TAS scores were highly correlated with negative effect (SCL-90-R anxiety & depression subscales) in ED patients. | Y | Y | Y | Y | 14 |
| Kühnpast et al. (55) | Y | Y | Y | Y | Y | N | Y | N | Y | Age, BMI, education, sex | Y | Y | N | N | Y | Y | N (Too small sample?) | Y | 12 |
| Lule et al. (56) | Y | Y | Y | Y | Y | N | Y | Y | Y | Age, education, cultural background, sex | N | Y | N | TAS-26 scores significantly positively correlated with EDQ and EDI subscale scores, and negatively correlated with BMI. | Y | Y | N (Too small sample?) | Y | 13 |
| Marchesi et al. (23) | Y | Y | Y | Y | Y | N | Y | Y | N | Age, sex | Y | Y | Significant differences between ED and controls in TAS-20 scores persisted after controlling for age and education, however after controlling for anxiety and depression, these differences disappeared. | Age and TAS-20 scores were significantly correlated. | Y | Y | Y | Y | 15 |
| Matsumato et al. (57) | Y | Y | Y | Y | Y | N | Y | Y | Y | Age, sex | Y | Y | N | N | Y | Y | Y | Y | 14 |
| Miyake et al. (58) | Y | Y | Y | Y | Y | N | Y | Y | Y | Sex | Y | Y | N | Unpleasantness ratings of words related to interpersonal relationships were negatively correlated with TAS-20 scores in AN. Significant correlations between TAS scores and BOLD responses in various brain regions were found. | Y | Y | Y | Y | 15 |
| Montebarocci et al. (59) | Y | Y | Y | Y | Y | N | Y | N | Y | Age, education, sex | Y | Y | Controlling for anxiety and depression, differences between groups in TAS scores were no longer significant. | TAS-20 scores were significantly positively correlated with cognitive subscales of the Bermond Vorst Alexithymia Questionnaire (BVAQ), but correlations with affective subscales were not signficant. | N | Y | N (Too small sample?) | Y | 13 |
| Nandrino et al. (60) | Y | Y | Y | Y | Y | N | Y | Y | Y | Age, sex, education | Y | Y | N | N | N | Y | N (Too small sample?) | Y | 12 |
| Nandrino et al. (61) | Y | Y | Y | Y | Y | N | Y | Y | N | Age, sex, education | N | Y | N | N | Y | Y | Y | Y | 12 |
| Parling et al. (62) | Y | Y | Y | Y | Y | N | Y | Y | Y | Age | Y | Y | When controlling for education and marital status, the significant group difference in TAS-20 scores remained. However, after controlling for depression or anxiety, the difference in TAS-20 scores disappeared. | No significant correlation between the TAS-20 and the LEAS. TAS-20 significantly correlated with depression and anxiety in both groups, and also with perfectionism in HCs only. | Y | Y | Y | Y | 16 |
| Pinaquy et al. | Y | Y | Y | Y | Y | N | Y | Y | Y | Age, BMI, education, SES, sex | Y | Y | N | TAS-20 scores significantly positively correlated with depression in the non-BED group only. TAS-20 score was a significant predictor of emotional eating in the BED group only. | Y | Y | Y | Y | 15 |
| Pollatos et al. (63) | Y | Y | Y | Y | Y | N | Y | Y | N | Age, sex, education | N | Y | N | TAS-20 scores were significantly negatively correlated with accuracy in identifying neutral faces. | N | Y | N (Too small sample?) | Y | 11 |
| Pollatos et al. (64) | Y | Y | Y | Y | Y | N | Y | Y | N | Age, sex, education | N | Y | N | TAS-20 subscale scores were significantly positively correlated with interoceptive awareness. | N | Y | Y | Y | 12 |
| Pollatos & Georgiou | Y | Y | Y | Y | Y | N | Y | N | N | Age, education, BMI | Y | Y | N | N | N | Y | Y | Y | 11 |
| Rommel et al. (40) | Y | Y | Y | Y | Y | N | Y | Y | Y | Sex | Y | Y | N | There were no significant correlations between TAS-20 and LEAS scores in either group. | Y | Y | Y | Y | 15 |
| Schmidt et al. (65) | Y | Y | Y | Y | N | N | Y | Y | N | N | Y | Y | N | TAS scores did not correlate with BMI, body shape concerns, or bulimic symptoms. | Y | Y | Y | Y | 12 |
| Sexton et al. (66) | Y | Y | Y | Y | N | N | Y | Y | Y | N | Y | Y | After controlling for depression, significant differences in TAS total scores between AN-BP and HCs disappeared, however AN-R still scored significantly higher than HCs. | Depression accounted for the largest proportion of variance in TAS total scores (21.9%), followed by presence of avoidant personality disorder (13%). A regression model including EDI-2 subscales showed that Interpersonal distrust explained a significant amount (45.8%) of variance in TAS total scores. | Y | Y | N (Too small sample?) | Y | 13 |
| Speranza et al. (41) | Y | Y | Y | Y | Y | N | Y | Y | N | Age, sex, professional status | Y | Y | N | TAS scores significantly positively correlated with depression and interpersonal dependency. Path analysis in all clinical groups showed that alexithymia predicted 2 dimensions of the depressive experiences questionnaire (DEQ): self-criticism and anaclitic, which predicted dependency. | Y | Y | Y | Y | 14 |
| Speranza et al. (67) | Y | Y | Y | Y | Y | N | Y | Y | N | Age, SES | Y | Y | After controlling for depression, BMI, and age, significant differences between patients and HCs persisted only for BN vs. HCs on TAS DIF & EOT factors, and for AN-R vs. HCs on TAS DDF factor (i.e., differences in TAS total scores were no longer significant). Inpatients did not show higher TAS scores than outpatients. | TAS total are DIF factor significantly positively correlated with depression and the self-criticism personality dimension of the DEQ in both AN & BN, and with the dependent personality dimension of the DEQ in AN only. | Y | Y | Y | Y | 15 |
| Strigo et al. (36) | Y | Y | Y | N | Y | N | Y | N | Y | Age, race, BMI, sex | Y | Y | N | TAS scores significantly positively correlated with right anterioir insula activty during pain anticipation in the rec AN group only. | Y | Y | N (too small sample and recovered AN only?) | N | 12 |
| Sureda et al. (68) | Y | Y | Y | Y | Y | N | Y | Y | Y | Age, education, sex | Y | Y | N | TAS scores were not related to age, age of illness onset, or duration of illness. TAS total scores significantly correlated with Jenkins Activity Survey (JAS) speed/impatience subscale in both groups. | Y | Y | Y | Y | 15 |
| Svaldi et al. (38) | Y | Y | Y | Y | Y | N | Y | Y | Y | Sex | N | Y | N | There were no significant correlations between TAS scores and desire to binge. | Y | Y | Y | Y | 14 |
| Taylor et al. (69) | Y | Y | Y | Y | N | N | Y | Y | N | Age, education, sex | Y | Y | N | TAS scores positively correlated with the following EDI subscales: ineffectiveness, interpersonal distrust, interoceptive awareness, and maturity fears in the AN group. In the matched HC group, only interpersonal distrust significantly correlated with TAS score. | Y | Y | Y | Y | 13 |
| Tchanturia et al. (70) | Y | Y | Y | Y | Y | N | Y | Y | Y | N | Y | Y | When depression was controlled for, the relationship between TAS and social anhedonia remained significant | TAS scores were significantly positively correlated with social anhedonia scores. | Y | Y | Y | Y | 15 |
| Torres et al. (71) | Y | Y | Y | Y | Y | N | Y | Y | Y | Age, education, sex | Y | Y | N | N | Y | Y | Y | Y | 14 |
| Torres et al. (72) | Y | Y | Y | Y | Y | N | Y | Y | Y | Age, education, sex, SES | Y | Y | Depression was the only significant predictor of variance in TAS scores in AN. | In AN, TAS scores significantly correlated with depression, but not age, BMI, illness and treatment duration, or medication use. | Y | Y | Y | Y | 16 |
| Troop et al. (73) | Y | Y | Y | Y | N | N | Y | Y | N | Sex | Y | Y | When controlling for age and social class, differences between groups on TAS scores largely remained significant. | N | Y | Y | Y | Y | 13 |
| Zeeck et al. (39) | Y | Y | Y | Y | N | N | Y | Y | N | Age, education, sex | Y | Y | When controlling for overall psychopathology, depression, and level of education, differences in TAS scores between groups remained significant. | Intensity of overall emotional experience was positively significantly correlated with TAS scores. Positive emotions correlated negatively and negative emotions correlated positively with TAS scores. However these correlations were not significant when controlling for general psychopathology and depression. TAS scores and general psychopahtology were positively correlated. | Y | Y | Y | Y | 14 |
| Zonnevylle-Bender et al. (74) | Y | Y | Y | Y | Y | N | Y | Y | Y | Age, education, SES | Y | Y | N | N | Y | Y | Y | Y | 14 |
| Zonnevylle-Bender et al. (75) | Y | Y | Y | N | Y | N | Y | Y | Y | Age, sex | Y | Y | After controlling for depression, difference in TAS-20 scores between groups remained significant. | TAS-20 scores significantly positively correlated with depression. | Y | Y | Y | Y | 15 |
